# Elevated Circular RNA PVT1 Promotes Eutopic Endometrial Cell Proliferation and Invasion of Adenomyosis via miR-145/Talin1 Axis

**DOI:** 10.1155/2021/8868700

**Published:** 2021-02-27

**Authors:** Yi-Yi Wang, Hua Duan, Sha Wang, Yong-Jun Quan, Jun-Hua Huang, Zheng-Chen Guo

**Affiliations:** ^1^Department of Minimally Invasive Gynecologic Center, Beijing Obstetrics and Gynecology Hospital, Capital Medical University, Beijing 100006, China; ^2^Department of Urology, Beijing Tongren Hospital, Capital Medical University, Beijing 100730, China

## Abstract

Several theories on the origin of adenomyosis (ADS) have been proposed, of which the most widely accepted is the fundamental pathogenic role of uterine eutopic endometrium. Emerging evidence suggests that circular RNAs participate in the multiple tumorgenesis. The vital importance of circular RNA PVT1 (circPVT1) in the pathological progress like malignancies has been well documented. Nevertheless, its underlying correlation with ADS remains elusive yet. The purpose of this study was to investigate the expression pattern, regulatory effect, and internal mechanism of circPVT1 in ADS. qRT-PCR was performed to detect the relative mRNA expression of circPVT1, miR-145, and Talin1 in ADS endometrial tissue and cells. The protein level of Talin1 was measured by Western blot and immunochemistry. Immunofluorescence was used to identify the primary endometrial epithelial and stromal cells. circPVT1 knockdown in vitro was achieved by transfecting with specific lentivirus vector CCK-8, and colony formation assays were utilized to assess cell proliferation; meanwhile, the transwell assay was employed for evaluating cell invasion ability. By conducting bioinformatics, dual-luciferase reporter assay, or RNA immunoprecipitation (RIP) experiment, the interaction between miR-145 and circPVT1 or Talin1 was verified. Rescue experiments further determined the regulatory effect of circPVT1/miR-145/Talin1 axis. We found both circPVT1 and Talin1 were markedly upregulated in ADS endometrial tissue and cells, whereas miR-145 was decreased. Elevated expression of circPVT1 was closely related to the severity of dysmenorrhea, menorrhagia, and uterine enlargement of patients with ADS. Knockdown of circPVT1 inhibited adenomyotic epithelial and stromal cell proliferation and invasion. Further mechanistic experiments revealed that circPVT1 negatively regulated miR-145 through serving as a molecular sponge. And the facilitating effect of circPVT1 was partially reversed by miR-145. Talin1 was demonstrated to be a down target of miR-145 and indirectly affected by circPVT1. Our findings unveiled that enhanced circPVT1 may be involved in the pathogenesis of ADS via stimulating endometrial cell proliferation and invasion. The establishment of circPVT1/miR-145/Talin1 pathway might present a novel therapeutic insight for ADS.

## 1. Introduction

Adenomyosis (ADS), typically characterized by ingravescent dysmenorrhea, menorrhagia, and female subfertility, is a common benign and refractory gynecological disorder. Although heavy attention has been paid, it remains an enigmatic disease for lacking in adequate understanding of either its etiology or pathogenesis. Among numerous hypotheses, the invagination theory is most widely accepted [1], which insists that ADS should be distinguished by the presence of aberrant growth and nonmalignant invasion of bioactive endometrium embedded within the myometrium. Furthermore, previous studies have also considered changes in the biological features of eutopic epithelial and stromal cells, such as enhancement of cell proliferation, invasiveness, and dynamics that might be closely related to the occurrence and progression of adenomyosis [2, 3].

Circular RNAs (circRNAs) are a new class of endogenous noncoding RNAs, deriving from exons and/or introns. They are featured as higher circulating stability, tissue specific, and abundant expression, which endow circRNAs with more stable biological functions and become potential candidate biomarkers for disease diagnosis and prognosis [4]. Indeed circRNAs play different and important roles in the onset and development of human carcinomas by regulating target genes in genetic or epigenetic level. However, to our best knowledge, whether circRNAs are involved in ADS is largely unknown.

Circular RNA PVT1 (circPVT1), derived from the known cancer susceptibility locus PVT1 exon-3, in chromosome 8q24, is an oncogenic noncoding RNA with emerging clinical importance [5]. It has been revealed that circPVT1 could be involved various human diseases as the competitive endogenous RNA (ceRNA). In particular, several studies have implied that circPVT1 facilitated cancer cell proliferation and invasion through binding to microRNAs (miRNAs) and regulating the target genes, thus leading to tumor growth and metastasis [6, 7]. Of note, adenomyotic endometrium also presents biological features similar to carcinomas. In the present study, we first provide evidence on the overexpression of circPVT1 in adenomyosis, both in the eutopic endometrial tissue and cells. By further performing bioinformatics analysis and experimental assays, miR-145/Talin1 axis was identified as a potential molecular pathway involved in circPVT1 regulating aberrant proliferation, invasion, and infiltration of endometrium. As the first systematic report on the correlation between circRNAs and ADS, our study may suggest a novel theoretical basis for elucidating the pathogenesis of ADS.

## 2. Materials and Methods

### 2.1. Clinical Sample Collection

Clinical tissue samples, including paired ADS eutopic and ectopic endometrium, as well as normal uterine endometrium, were collected from patients who had undergone a transabdominal, transvaginal, or laparoscopic hysterectomy at Beijing Obstetrics and Gynecology Hospital, Capital Medical University from February 2017 to October 2019. There were 45 patients with ADS recruited in the study group. The control group consisted of 40 women without histopathological evidence of ADS, and they underwent hysterectomy for early-stage ovarian cancer or cervical cancer. All of participants were premenopausal with regular menstrual cycles and at proliferative or secretory phase during the procedure. Any participant with indication of concomitant endometriosis, endometrial pathology or malignancy, history of hormone therapy, or intrauterine device placement within 3 months preoperatively was all excluded [8]. The study was approved by the local ethics committee (No. 2016-KY-012-02), and all participants gave their informed consent for the research.

The removed endometrium samples were partly immersed into 0.9% sterile saline containing penicillin and streptomycin and immediately sent to the laboratory for subsequent primary cell culture, whereas the rest were frozen at -80°C for the following RNA and protein extraction.

### 2.2. Cell Culture and Identification

The primary adenomyotic eutopic endometrial epithelial and stromal cells (Eu_EEC and Eu_ESC, respectively) were isolated from the eutopic endometrium samples and cultured in vitro according to our previous protocol with minor modification [9]. The separated endometrial tissue was minced into 1-2 mm^3^ after being rinsed 2-3 times with PBS (Gibco, USA) to remove impurities and most blood cells. Add 0.2% type I collagenase (Sigma, USA) containing 0.005% deoxyribonuclease (Invitrogen, USA) to fully digest for around 1 hour at 37°C. Afterwards, DMEM/F12 (Hyclone, USA) containing 12.5% FBS (BD, USA) was added and subsequently filtered through the 100 *μ*m cell strainer, getting the cell suspension without digested tissue and mucosa. After the filtrate was centrifuged (500 rpm, 3 min), passing through 40 *μ*m cell strainer, the obtained precipitate was centrifuged again to isolate Eu_EEC; meanwhile, the secondary filtrate was centrifuged twice (1200 rpm, 3 min), and the final cell suspension was the Eu_ESC. Both Eu_EEC and Eu_ESC were seeded and cultured with DMEM/F12 containing 12.5% FBS, 1% antibiotic penicillin, and streptomycin in an incubator (37°C, 5% CO_2_).

The primary Eu_EEC and Eu_ESC were observed after incubating 24-48 h (Figure S1A).

On the basis of previous protocols reported, we performed immunofluorescence assay to identify the specific markers of Eu_EEC and Eu_ESC, respectively. First of all, cells were observed and photographed under an inverted microscope. When cells on slides reached 70-80% confluence, they were fixed with 4% paraformaldehyde for 20 min at room temperature. After removing the cell debris with PBS, we subsequently used 0.2% Triton-X-100 to permeabilize cells and 1% bovine serum albumin (BSA) for blocking (37°C, 30 min) in succession. The Eu_EEC and Eu_ESC were incubated with the primary antibodies against pan-cytokeratin (mouse, 1 : 100, Santa Cruz Biotechnology, USA) and Vimentin (rabbit, 1 : 50, Boster Biological Technology, China) overnight at 4°C, respectively. Afterwards, the Eu_EEC was incubated with the secondary antibody, donkey anti-mouse IgG Alexa Fluor 555 (1 : 500, Beyotime, China), while the Eu_ESC was incubated with goat anti-rabbit IgG Alexa Fluor 488 (1 : 500, Beyotime, China) for 30 minutes at 37°C. Then, DAPI was used for nuclear counterstaining. The images were observed and captured under a fluorescence microscope (Figure S1B).

### 2.3. Cell Transfection

The short hairpin RNA (sh-RNA) against circPVT1 (sh-circPVT1) and nontargeting sequences expressing a scramble RNA as negative control (sh-NC) were constructed in GV248-hU6-Ubiquitin-EGFP-IRES-puromycin lentivirus vector (GeneChem Co., Shanghai, China). Meanwhile, the lentivirus vector containing circPVT1 overexpression plasmid (GV367-circPVT1) or its negative control GV367 was also synthesized and cloned by GeneChem. When cells in 6-well plates reached 50-60% confluence, the four plasmids sh-circPVT1, sh-NC, GV367-circPVT1, and GV367 were transfected into the Eu_EEC and Eu_ESC by using a lipofectamine 3000 (Invitrogen, USA), according to the manufacturer's instructions. Forty-eight hours after transfection, 1 *μ*g/ml puromycin was added to select the transfected positive cells; subsequently, quantitative reverse transcription PCR (qRT-PCR) was performed to evaluate the transfection efficiency and further ensured that remaining cells were actually transfected.

### 2.4. RNA Isolation and Quantitative Reverse Transcription PCR (qRT-PCR)

Total RNA from endometrial tissue samples and cells was extracted using RNA isoPlus (Takara, Bio Inc, Japan). For the reverse transcription reaction of circPVT1, miR-145, and Talin1 mRNA, the PrimeScript RT Reagent Kit (Takara, Japan) was used to synthesize cDNA. The subsequent quantitative PCR reaction was performed following the protocol of a SYBR Green PCR Kit (Takara, Japan) through an ABI 7500 PCR system (Applied Biosystems, Grand Island, USA). GAPDH, U6, and *β*-actin were selected as the reference gene for normalizing the expression of target gene circPVT1, miR-145, and Talin1, respectively. And the results were calculated and analyzed with 2^-*ΔΔ*ct^ method. All the specific primers in the study were synthesized by Sangon Biotech (Shanghai, China) as listed in Table S1. The experiment was repeated for 3 times.

### 2.5. Immunohistochemistry

Immunohistochemistry for Talin1 in ADS and normal uterine endometrium was performed as our previous study described [37]. Briefly, the paraffin-embedded tissue samples were cut into 4 *μ*m sections. Thereafter, routine procedures including deparaffinizing, rehydrating, and incubating with 3% hydrogen peroxide were performed. For antigen retrieval, sections were boiled in 10 mmol/L citrate buffer and cooled at room temperature. After blocking the nonspecific binding with 3% BSA (Servicebio, China) for 30 min at 37°C, the sections were subsequently incubated with Talin1 primary rabbit polyclonal antibody (1 : 100, Abcam, UK) overnight at 4°C. Then, a horseradish peroxidase-conjugated goat anti-rabbit secondary antibody was added for 1-hour incubation. Next, the sections were rinsed with PBS and treated with 3′-diaminobenzidine tetrahydrochloride. After being counterstained with hematoxylin and eosin, all the sections were eventually mounted, examined, and imaged.

### 2.6. Western Blot

Total proteins were extracted with RIPA lysis buffer (Sigma, St. Louis) and quantified using BCA Protein Assay Kit (Beyotime, China). Then, equal proteins were subjected to 10% SDS-PAGE and transferred onto a PVDF membrane. After being blocked in 5% skim milk containing 1 × TBST (Solarbio, China) at room temperature for 1 h, the membrane was incubated with anti-Talin1 antibody (1 : 500, Abcam, UK) and actin (1 : 1000, Cell Signaling Technology, USA) overnight at 4°C. Getting washed with TBST, the secondary antibodies were used for incubation. Finally, the immunoreactive bands were detected with Chemiluminescent HRP Substrate (Merck Millipore) in a Bio-Rad imaging system (Hercules, CA, USA).

### 2.7. Dual-Luciferase Reporter Assay

The Circinteractome database or TargetScan database was employed for the prediction of the putative binding sites between miR-145 and circPVT1 or Talin1. The Eu_EEC and Eu_ESC Cells were seeded into 24-well plates (5 × 10^5^/well). The fragment of circPVT1-WT3′UTR, circPVT1-MT3′UTR, Talin1-WT3′UTR, and Talin1-MT3′UTR were inserted into the pGL3 vector. Then, the reporter vectors and miR-145 mimics or its negative control miR-145 NC were cotransfected into cells. After 48 hours, the relative luciferase activity was determined and analyzed by normalizing the firefly against Renilla luciferase activity using the dual-luciferase reporter assay kit (Promega).

### 2.8. RNA Immunoprecipitation (RIP)

To further verify the relationship between circPVT1 and miR-145, we performed the RNA-binding protein immunoprecipitation assay in the HEK-293T cells using the Magna RIP Kit (Millipore, USA) together with the AgO2 antibody (Abcam, USA). Following the manufacturer's instructions, cells were lysed via RIP lysis buffer and then the cell lysate was incubated in the RIP buffer harboring magnetic beads conjugated with anti-AgO2 or anti-IgG antibodies. Subsequently, to recover the AgO2 antibody, the magnetic bead protein was used for incubation. At last, coprecipitated RNAs were measured through qRT-PCR analysis.

### 2.9. Cell Proliferation Assay

72 hours after transfection, the cell viability of Eu_EEC and Eu_ESC was examined with the Cell-Counting Kit-8 (CCK-8, Dojindo, Technology Co., Ltd). Eu_EEC and Eu_ESC cells transfected with different plasmids were seeded in 96-well plates at 5000 cell per well and cultured for 0 h, 24 h, 48 h, 72 h, or 96 h. Then, 10 *μ*l/well CCK-8 reagent was added at indicated time points, and the corresponding cells were incubated for another 4 hours at 37°C. Eventually, the absorbance at 450 nm was measured with a microplate reader (Bio-Rad, USA).

### 2.10. Plate Colony Formation Assay

The Eu_EEC and Eu_ESC were seeded in 6-well cell plates at 1000 cells/well density after being transfected for 72 hours. Thereafter, cells were incubated for two weeks during which the medium was refreshed every 2-3 days. After finishing incubation, the cells were fixed with 4% paraformaldehyde and were stained with 0.1% crystal violet for 20 minutes at room temperature. Finally, the number of visible colonies was counted under a microscope.

### 2.11. Cell Invasion Assay

The cell invasion assay was performed using 24-well Transwell plates (8.0 *μ*m pore size with polycarbonate membrane, Costar) coated with Matrigel (BD Bioscience, 1 : 8 dilution) in the upper chamber. Briefly, the posttransfected cells were seeded into the upper chamber and cultured with 200 *μ*l serum-free DMEM/F12, while the bottom chamber contained 600 *μ*l of medium with 12.5% FBS as a chemoattractant. After 48 h of incubation, cells on the lower side of the chamber were fixed with 4% paraformaldehyde and stained with 0.1% crystal violet for 20 minutes, respectively. Finally, cells that had invaded into the lower surface were captured and evaluated in 5 randomly selected areas.

### 2.12. Statistical Analysis

The collected results were processed and analyzed using the SPSS 23.0 and GraphPad Prism 8.0 software. All data were presented and evaluated as mean ± standard deviation (SD). Analysis of variance, independent-sample *t*-tests, and *λ*^2^ tests were performed to assess the difference, as appropriate. A *P* value less than 0.05 was considered to indicate statistical significance.

## 3. Results

### 3.1. circPVT1 Was Upregulated in Human ADS and Associated with Clinical Characteristics

**Figure 1:**
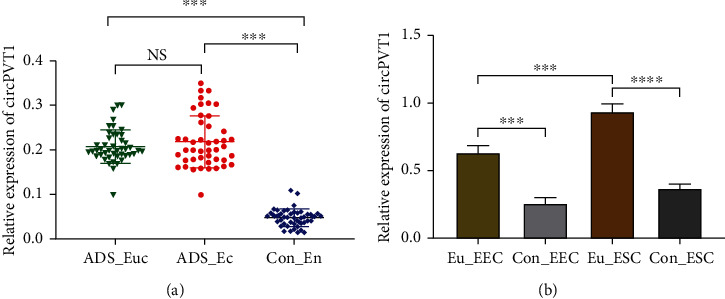
circPVT1 was upregulated in human ADS endometrial tissue and cells. (a) The relative expression of circPVT1 was elevated in adenomyotic eutopic endometrium (ADS_Euc, *n* = 45) and ectopic endometrium (ADS_Ec, *n* = 45), as detected by qRT-PCR and normalized against unpaired normal uterine endometrium (Con_En, *n* = 40). (b) qRT-PCR analysis of circPVT1 in ADS and normal uterine endometrial cells. Four types of primary cells are as follows: Eu_EEC, adenomyotic eutopic endometrial epithelial cell; Eu_ESC, adenomyotic eutopic endometrial stromal cell; Con_EEC, normal uterine endometrial epithelial cell as control; Con_ESC, normal uterine endometrial stromal cell as control. At least triplicate independent experiments were performed. All data were presented as mean ± SD. ^∗^*P* < 0.05, ^∗∗^*P* < 0.01, ^∗∗∗^*P* < 0.001, and ^∗∗∗∗^*P* < 0.0001; NS: no significance.

To investigate the potential role of circPVT1 in ADS, we firstly performed qRT-PCR to detect its expression level. As presented in Figure 1(a), circPVT1 expressed significantly higher both in eutopic (*n* = 45) and in ectopic (*n* = 45) endometrium of ADS than that of the control group (*n* = 40). However, there was no statistical difference between the ADS eutopic endometrium (ADS_Euc) and ADS ectopic endometrium (ADS_Ec) groups. Correspondingly, we also observed that circPVT1 was also upregulated in ADS eutopic endometrial epithelial and stromal cells (Eu_EEC and Eu_ESC) compared with the normal uterine endometrial cells (Figure 1(b)). In addition, further analysis of the relationship between circPVT1 expression and clinical factors of ADS indicated that increased circPVT1 was positively correlated with the severity of dysmenorrhea^a^ and menorrhagia^b^ (*r*_*a*_ = 0.371, *P*_*a*_ = 0.016 and *r_b_*= 0.221, *P*_*b*_ = 0.039) which could be validated through the VAS and PBAS scores, respectively. From our study, the ADS uterine larger than 200 mm^3^ and the diffuse type were more susceptible to the elevated circPVT1 expression (Table 1).

### 3.2. Knockdown of circPVT1 Repressed ADS Eutopic Endometrial Epithelial and Stromal Cell Proliferation and Invasion

**Figure 2:**
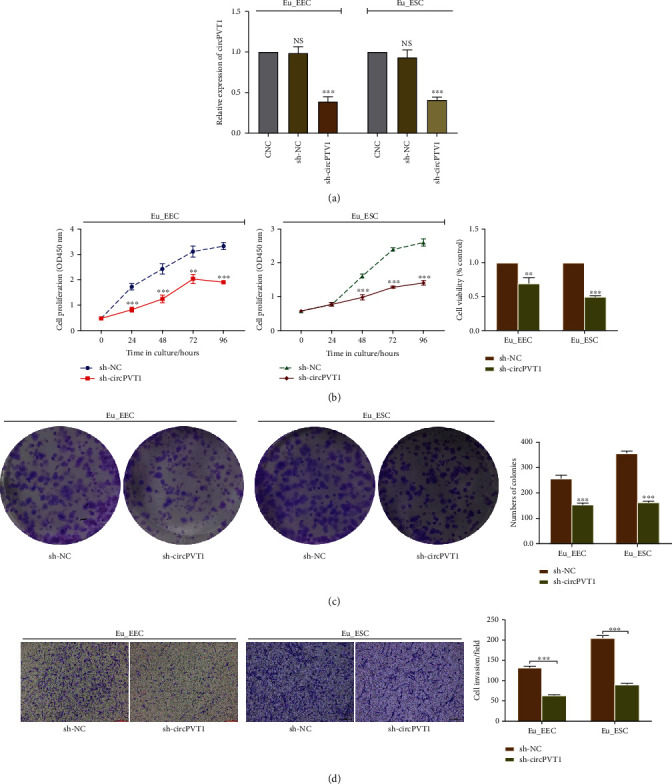
Knockdown of circPVT1 repressed ADS eutopic endometrial epithelial and stromal cell proliferation and invasion. (a) Silencing efficiency of circPVT1 in Eu_EEC and Eu_ESC cells was determined using qRT-PCR after transfecting with circPVT1 sh-RNA (sh-circPVT1) or its negative control vector (sh-NC); the CNC group represented cells without any transfection. (b) Cell Counting Kit-8 (CCK-8) assay was performed to assess the effect of sh-circPVT1 on the cell proliferation. The histogram illustrated the cell viability of Eu_EEC and Eu_ESC determined by the CCK-8 assay at 96 hours posttransfection. (c) Effect of circPVT1 knockdown on the colony formation ability of Eu_EEC and Eu_ESC. (d) Effect of circPVT1 knockdown on the cell invasion capacity was examined by the transwell assay. The number of invasive cells was counted after 72 hours. At least triplicate independent experiments were performed. All data were presented as mean ± SD. ^∗^*P* < 0.05, ^∗∗^*P* < 0.01, ^∗∗∗^*P* < 0.001, and ^∗∗∗∗^*P* < 0.0001; NS: no significance.

In view of the overexpression of circPVT1 in ADS endometrium and cells, we speculated that circPVT1 may serve an oncogenic role in the development of ADS. To further explore the biological effect of circPVT1 on ADS, we transfected the Eu_EEC and Eu_ESC with sh-NC or sh-circPVT1 to silence its expression specifically. And qRT-PCR was performed to verify the interference efficiency (Figure 2(a)). As illustrated in Figures 2(b) and 2(c), both Eu_EEC and Eu_ESC cell growth got inhibited once circPVT1 was downregulated, represented by the decreased proliferation rate and less colony formation. Meanwhile, the results of transwell assay demonstrated that the invasion ability of Eu_EEC and Eu_ESC was notably weakened in the sh-circPVT1 group (Figure 2(d)). Therefore, these results implied that circPVT1 might promote ADS endometrial cell progression in vitro.

### 3.3. CircPVT1 Negatively Regulated miR-145 Serving as a Molecular Sponge

**Figure 3:**
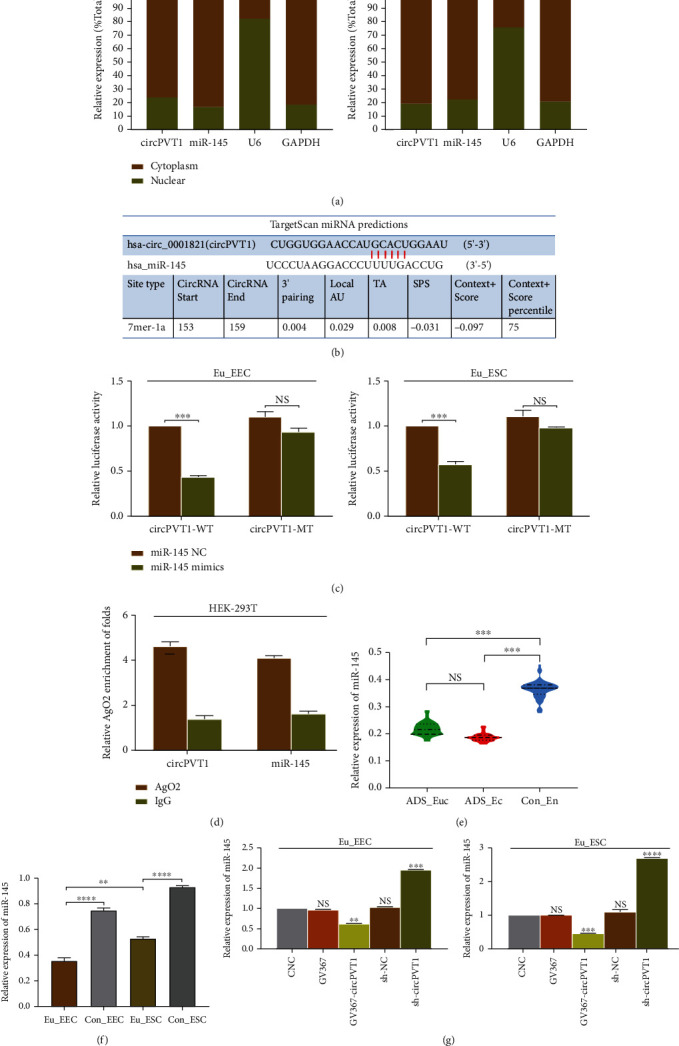
circPVT1 negatively regulated miR-145 serving as a molecular sponge. (a) The relative expression levels of circPVT1 and miR-145 in the cytoplasmic and nuclear fractions of Eu_EEC and Eu_ ESC were measured by qRT-PCR, respectively. GAPDH represented the cytoplasmic marker, and U6 was detected as the nuclear reference. (b) Bioinformatics analysis was utilized to predict the putative binding sites between circPVT1 and miR-145. (c) The relative luciferase activity of wide-type circPVT1 (circPVT1-WT) or mutant-type circPVT1 (circPVT1-MT) in the indicated cells cotransfected with miR-145 mimics or miR-145 negative control (miR-145 NC). (d) Cellular lysate of HEK-293T cell was collected and immunoprecipitated. The relative amount of circPVT1 or miR-145 bound to AgO2 antibody was detected using qRT-PCR. IgG was used as a negative control. (e) miR-145 expression levels were decreased in adenomyotic eutopic endometrium (ADS_Euc, *n* = 45) and ectopic endometrium (ADS_Ec, *n* = 45). (f) qRT-PCR detection of miR-145 in ADS and normal uterine endometrial cells. Four types of primary cells are as follows: Eu_EEC, adenomyotic eutopic endometrial epithelial cell; Eu_ESC, adenomyotic eutopic endometrial stromal cell; Con_EEC, normal uterine endometrial epithelial cell as control; Con_ESC, normal uterine endometrial stromal cell as control. (g) Relative expression of miR-145 in Eu_EEC and Eu_ESC cells treated with circPVT1 overexpression (GV367-circPVT1) or circPVT1 knockdown (sh-circPVT1), respectively. The CNC group represented cells without any transfection. At least triplicate independent experiments were performed. All data were presented as mean ± SD. ^∗^*P* < 0.05, ^∗∗^*P* < 0.01, ^∗∗∗^*P* < 0.001, and ^∗∗∗∗^*P* < 0.0001; NS: no significance.

Emerging evidence has shown that circPVT1 may function as a molecular sponge in cytoplasm, competitively binding to the target microRNA (miRNA) to lower its abundance and activity [10–12]. Firstly, a nucleocytoplasmic isolation assay demonstrated that circPVT1 was mainly located in the cytoplasm of Eu_EEC and Eu_ESC (Figure 3(a)), offering an essential prerequisite for its interaction with the downstream miRNA. Meanwhile, miR-145 was also found to be mostly enriched in cytoplasm. As Figure 3(b) presented, we found that miR-145 shared specific binding sequence with circPVT1 in the subsequent bioinformatics analysis (Circular RNA database and Human miRNA Disease database were searched). Furthermore, in the dual-luciferase reporter assay, the circPVT1-WT or circPVT1-MT vector was constructed and cotransfected into cells, which indicated that miR-145 mimics markedly reduced the luciferase activity of circPVT1 while displaying no effect on the mutant controls (Figure 3(c)). In addition, we also observed circPVT1 and miR-145 were both much more enriched in AgO2 compared to IgG (Figure 3(d)). To investigate whether miR-145 could be influenced by circPVT1 in ADS, the decreased expression of miR-145 was observed in ADS endometrial tissue and cells (Figures 3(e) and 3(f)), which was negatively correlated with circPVT1. Then, overexpression of circPVT1 exhibited by transfection of GV367-circPVT1 led to a significant lower level of miR-145 in Eu_EEC and Eu_ESC and vice versa (Figure 3(g)). Taken together, it was verified that in ADS, circPVT1 might exert inhibitory effect on miR-145 through the intrinsic sponging mechanism.

### 3.4. miR-145 Rescued the Promoting Effect of circPVT1 on Adenomyotic Eutopic Endometrial Cells

**Figure 4:**
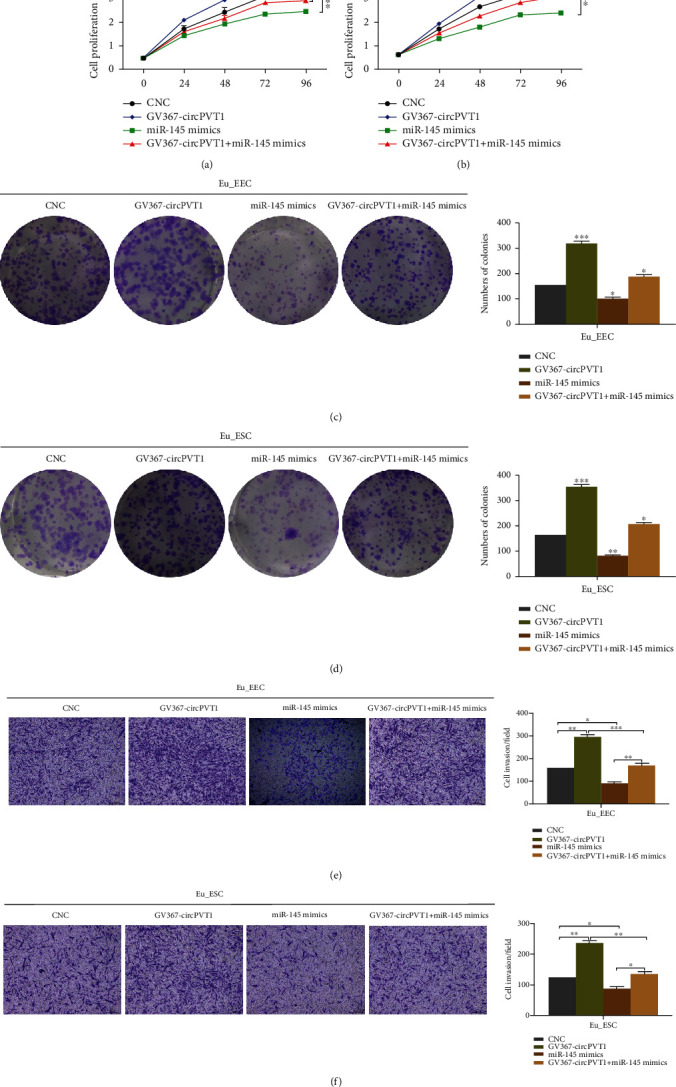
miR-145 rescued the promoting effect of circPVT1 on adenomyotic eutopic endometrial cells. (a, b) The proliferation capacity of Eu_EEC and Eu_ESC was evaluated by Cell-Counting Kit-8 (CCK-8) assays after transfection. (c, d) Colony formation assays were performed to assess the colony formation ability of Eu_EEC and Eu_ESC transfected with GV367-circPVT1, miR-145 mimics, or GV367-circPVT1+miR-145 mimics. (e, f) Different effects of GV367-circPVT1, miR-145 mimics, and GV367-circPVT1+miR-145 mimic treatment on the cell invasion of Eu_EEC and Eu_ESC. The CNC group represented cells without any transfection; GV367-circPVT1, circPVT1 overexpression; miR-145 mimics, miR-145 upregulation; GV367-circPVT1+miR-145 mimics, cells cotransfected with circPVT1 overexpression and miR-145 upregulation vectors. At least triplicate independent experiments were performed. All data were presented as mean ± SD. ^∗^*P* < 0.05, ^∗∗^*P* < 0.01, ^∗∗∗^*P* < 0.001, and ^∗∗∗∗^*P* < 0.0001; NS: no significance.

In order to further clarify how miR-145, as a target of circPVT1, affected the onset and progress of ADS, we performed the rescue experiment through cotransfection of GV367-circPVT1 overexpression vector and miR-145 mimics. The results indicated that miR-145 decreased cell viability while circPVT1 alone had the most significant acceleration on the cell proliferation. However, data from the cotransfection group demonstrated that circPVT1's stimulative effect on the multiplication capacity of Eu_EEC and Eu_ESC got partially abolished by miR-145 (Figures 4(a)–4(d)). Similarly, Figures 4(e) and 4(f) illustrated that miR-145 also abrogated the cell invasion induced by circPVT1 to some extent. Anyway, whether in Eu_EEC or in Eu_ESC, the facilitating effect of circPVT1 on cell proliferation and invasion ability would be weakened or reversed by miR-145. These results provided further evidence to prove that circPVT1 might be involved in ADS via competitively binding to miR-145.

### 3.5. Talin1, a Downstream Target of miR-145, Was Positively Affected by circPVT1

**Figure 5:**
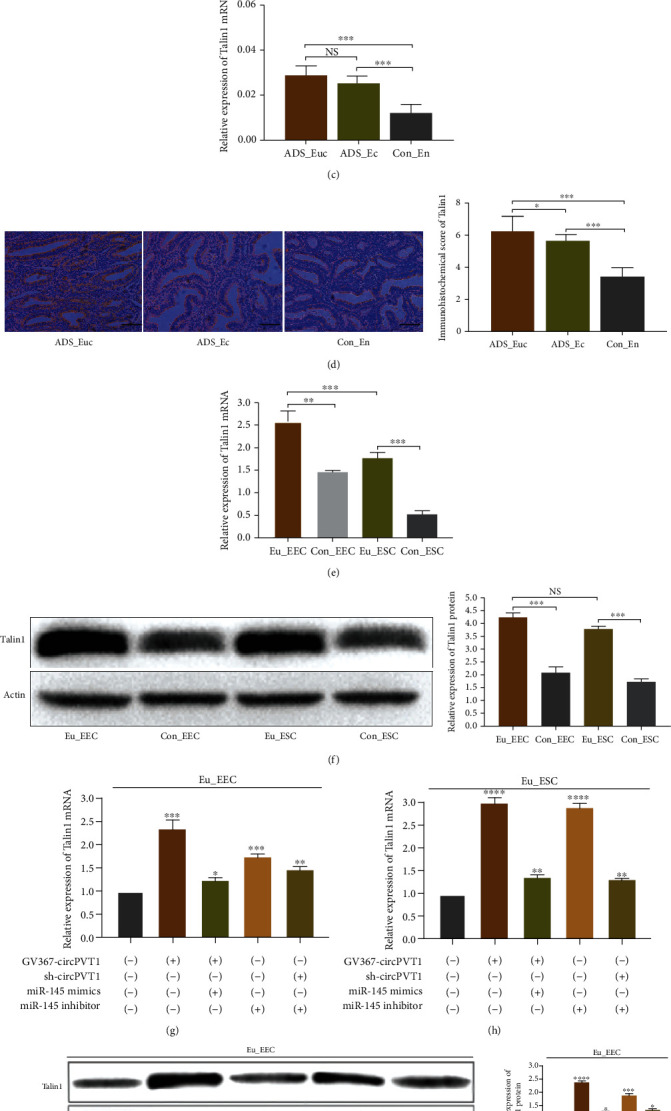
Talin1, a downstream target of miR-145, was positively affected by circPVT1. (a) The predicted binding sites between miR-145 and Talin1 through bioinformatics. (b) Wide-type Talin1 (Talin1-WT) or mutant-type Talin1 (Talin1-MT) luciferase reporter was cotransfected into Eu_EEC and Eu_ESC with miR-145 mimics or its negative control (miR-145 NC). The relative luciferase activity was determined. (c, d) The relative mRNA or protein levels of Talin1 in ADS (*n* = 45) and normal uterine endometrium (*n* = 40) were evaluated by qRT-PCR and immunohistochemical staining, respectively. The magnifications of the micrographs were ×400. Scale bars represented 5 *μ*m. (e, f) The relative mRNA or protein levels of Talin1 in ADS and normal uterine endometrial cells were detected by qRT-PCR and Western blot. (g–j) circPVT1 overexpression or miR-145 knockdown increased the mRNA and protein expression of Talin1 in Eu_EEC and Eu_ESC, whereas cotransfection with miR-145 mimics or sh-circPVT1 got Talin1 expression abolished. ADS_Euc, adenomyotic eutopic endometrium; ADS_Ec, adenomyotic ectopic endometrium; Con_En, the normal uterine endometrium as control; Eu_EEC, adenomyotic eutopic endometrial epithelial cells; Eu_ESC, adenomyotic eutopic endometrial stromal cells; GV367-circPVT1, circPVT1 overexpression; sh-circPVT1, knockdown of circPVT1; miR-145 mimics, miR-145 upregulation; miR-145 inhibitor, inhibition of miR-145. At least triplicate independent experiments were performed. All data were presented as mean ± SD. ^∗^*P* < 0.05, ^∗∗^*P* < 0.01, ^∗∗∗^*P* < 0.001, and ^∗∗∗∗^*P* < 0.0001; NS: no significance.

To investigate how miR-145 regulated the target gene expression at the transcriptional and posttranscriptional level, we performed the related bioinformatics analysis to predict the potential target genes of miR-145 in ADS, by which Talin1 was screened out (Figure 5(a)). Additionally, the results from the dual-luciferase reporter assay revealed that cotransfection of miR-145 and Talin1-WT vector notably inhibited the luciferase activity, indicating there were binding sites between miR-145 and Talin1 (Figure 5(b)). Then, as expected, we observed that Talin1 mRNA and protein levels were both elevated in ADS endometrium, as did its overexpression in Eu_EEC and Eu_ESC cells (Figures 5(c)–5(f)). After performing upregulation and interference intervention on the circPVT1 and miR-145, respectively, qRT-PCR and Western blot assay were utilized to detect the corresponding difference of Talin1 expression. The results showed that Talin1 mRNA would get increased accordingly once circPVT1 upregulated or miR-145 inhibited individually. In particular, the promoted effect on Talin1 was markedly abolished when sh-circPVT1 and miR-145 mimics were cotransfected into cells (Figures 5(g) and 5(h)). Meanwhile, the protein expression level of Talin1 was consistent with the results from qRT-PCR, exhibiting the similar variation tendency (Figures 5(i) and 5(j)). These findings implied that Talin1 was a target of miR-145, and circPVT1 might affect ADS endometrial cell function via modulating the miR-145/Talin1 axis. Eventually, to clearly summarize and explain the proposed circPVT1/miR-145/Talin1 regulatory pathway in the etiopathogenesis of ADS, we used a schema chart in Figure S2.

## 4. Discussion

So far, the exact pathogenesis of adenomyosis (ADS) has been largely a conundrum. However, theories on the aberrant changes of uterine eutopic endometrial biological function are the most widely accepted with robust evidence. Particularly, the active proliferation and enhanced invasion ability of endometrial epithelial and stromal cells, similar to the malignant tumor cells, have been considered as the key step to induce the ectopic implantation and infiltration of the endometrium inside myometrium, eventually leading to the onset of ADS [3, 13, 14]. Recently, more studies have revealed that noncoding RNAs, as one of the important methods of epigenetic modification, may be closely related to the occurrence of ADS [15, 16]. Circular RNAs (circRNAs), featured as covalently linked terminals and higher stability, are a novel class of noncoding RNAs. Abundant evidence suggested that circRNAs mostly regulate the target gene expression via the response element-mediated interaction with miRNAs, playing a vital role in cellular progress and tumorgenesis [17, 18]. Of note, circRNA profiles have been identified in eutopic endometrium of endometriosis (EMs), which shares a lot in common with ADS in the pathogenesis [19, 20]. And the upregulated circ_0004712 and circ_0002198 in EMs might be involved in fostering endometrial cell proliferation, migration, and invasion while inducing the cell apoptosis resistance [21]. Nevertheless, whether circRNAs can mediate ADS through affecting the biological function of endometrial cells, the related data remain extremely scanty.

In the present study, we unveiled that circPVT1, an oncogenic noncoding RNA with accumulating clinical importance, was significantly upregulated in ADS endometrial tissue and cells. As per available literature, circPVT1 was first detected in gastric cancer by Chen et al. [22], and its overexpression has been confirmed to be involved in the pathogenesis of various malignant conditions [10–12, 23, 24], including the osteosarcoma, non-small-cell lung carcinoma (NSCLC), glioblastoma, colorectal and liver cancer, multiple myeloma (MM), and acute lymphoblastic leukemia (ALL). Without exception, circPVT1 plays an oncogenic role in all of these malignancies, during which the positive effect on cell proliferation was the most common. Considering the accordant increased tendency in ADS, we speculated that potential association existed in its abnormal expression and clinical characteristics of patients with ADS. Especially, dysmenorrhea, menorrhagia, and uterine enlargement, as the typical manifestation of ADS, were all demonstrated to be related to higher circPVT1 level. These findings further proved that circPVT1 might participate in the occurrence and progress of ADS. And subsequent in vitro experiments identified that deletion of circPVT1 strikingly inhibited the proliferation and invasion ability of eutopic endometrial cells (Eu_EEC and Eu_ESC). That is, we first found and verified that elevated circPVT1 might facilitate faster growth of endometrial cells and stronger infiltration to the uterine myometrium by serving as an oncogene in ADS.

It has been reported that circPVT1was more stable and preferentially more cytoplasmic in cellular localization [5]. Emerging evidence indicated that circPVT1 might mostly function as competitive endogenous RNA (ceRNA) [25]. In our study, the nucleocytoplasmic separation assay showed that circPVT1 expressed much higher in the cytoplasmic fractions than that of nuclear. This was consistent with the previous studies, meaning that circPVT1 might regulate the target gene expression and affect cell function by sponging to miRNAs. Using the online bioinformatics analysis, we predicted the targeted binding sites between circPVT1 and miR-145 in ADS. Thereafter, the dual-luciferase reporter assay and RIP experiment both provided further confirmation on the speculation that circPVT1 could regulate miR-145 in a sequence-dependent manner in adenomyotic epithelial and stromal cells. Reviewing the literature, Zheng and Xu reported that circPVT1 contributed to the chemotherapy resistance of lung cancer via binding miR-145 specifically [26]. And the metastasis of colorectal tumor cells also got promoted through the interaction between circPVT1 and miR-145 [27]. Taken together, our results were strongly supported.

The latest studies have identified that various miRNAs exhibited differential expression in ADS, controlling a vast number of RNA transcripts to be involved in the cellular pathophysiological activities. MiRNA-10b was found to be able to directly target ZEB1 and PIK3CA, thus curbing adenomyotic epithelial cell invasiveness [28]. And our previous findings also suggested that Lin28B/miRNA-Let7a axis could affect the cell proliferation of ADS-junctional zone [9]. While there is abundant evidence on the vital role miR-145 exerts in the development of tumors, mostly proposed as a suppressor [29, 30], it remains a novel concept for ADS. According to our present study, miR-145 was decreased in adenomyotic endometrial tissue and cells, whose expression level was also negatively affected by circPVT1 in Eu_EEC and Eu_ESC. Furthermore, to functionally verify the role of miR-145 in ADS endometrial cell growth, we performed the rescue experiment in vitro, and observed that the facilitating effect of circPVT1 on cell proliferation got distinctly subdued by miR-145 mimics. Similarly, the invasive ability of adenomyotic Eu_EEC and Eu_ESC was also weakened when co-transfecting miR-145 mimics and GV367-circPVT1 vector (overexpression of circPVT1). Therefore, the circPVT1/miR-145 regulatory pathway was unveiled in the eutopic endometrial epithelial and stromal cells of ADS.

Talin1, a ubiquitous cytoskeletal protein locating at the cell-matrix attachment sites, mostly functions as the key regulator of integrin activation [31, 32]. As the previous studies implicated, Talin1 acts an essential role in stimulating the integrin-mediated signal transduction, thus affecting cell adhesion, proliferation, differentiation, migration, and invasion [33]. More evidence has demonstrated that Talin1 was overexpressed and involved in the progress of multiple human tumors [34–36]. In addition, Talin1 was also detected to be upregulated in the eutopic and ectopic endometrial glands of ADS [37]. However, in this study with limited sample size, the underlying mechanism of the relationship between Talin1 aberrant expression and the pathogenesis of ADS has not been elucidated. As expected, we also observed that Talin1 presented a significantly higher mRNA and protein level in adenomyotic endometrial tissue and cells than that of the control group. Moreover, the 3′UTR of Talin1 was demonstrated to be directly bound by miR-145. Based on the speculation that Talin1, as the downstream target of miR-145, might be related to the immoderate proliferation and invasion of Eu_EEC and Eu_ESC, we further detected the changes of Talin1 expression under the different interventions on circPVT1 or miR-145. Our results suggested that elevated circPVT1 increased the expression of Talin1 while the treatment with miR-145 mimics precisely showed the opposite effect and vice versa. That is, Talin1 was positively regulated by circPVT1 and negatively affected by miR-145. Taken together, we discovered that aberrant expression of circPVT1 could modulate the biological activity of ADS endometrial cells via miR-145/Talin1 axis. However, the detection and functional confirmation of circPVT1/miR-145/Talin1 pathway was merely performed in vitro. And our findings just focused on the adenomyotic endometrial tissue and cells, which still have limitations in fully clarifying the pathogenesis of ADS. Last but not least, further discussion on how Talin1 affected the occurrence and progress of ADS should not be ignored.

Collectively, our study unveiled that circPVT1 was aberrantly upregulated in human adenomyosis. It could facilitate the proliferation and invasion of ADS eutopic endometrial epithelial and stromal cells through miR-145/Talin1 regulatory axis, probably providing a novel diagnostic and therapeutic insight for ADS.

## Figures and Tables

**Table 1:** Correlation between circPVT1 expression in eutopic endometrial tissue and clinical features of patients with ADS (*n* = 45).

Variables	Case (*n* %)	Relative expression of circPVT1^∗^	*P* value
Age, years			0.689
<42	11 (24.4%)	0.217 ± 0.030	
≥42	34 (75.6%)	0.205 ± 0.004	
Gravidity			0.852
0	2 (4.4%)	0.193 ± 0.012	
1-2	24 (53.3%)	0.212 ± 0.037	
≥3	19 (42.3%)	0.209 ± 0.008	
Parity			0.533
0	4 (8.9%)	0.201 ± 0.017	
1	18 (40.0%)	0.225 ± 0.041	
≥2	23 (51.1%)	0.216 ± 0.015	
Menstrual phase			0.604
Proliferative	21 (46.7%)	0.197 ± 0.009	
Secretory	24 (53.3%)	0.203 ± 0.040	
Classification of ADS			0.041
Focal	9 (20.0%)	0.192 ± 0.006	
Diffuse	36 (80.0%)	0.273 ± 0.018	
PBAC scores			0.039
<100	14 (31.1%)	0.211 ± 0.018	
≥100	31 (68.9%)	0.304 ± 0.006	
VAS scores			0.016
0-3	3 (6.7%)	0.185 ± 0.032	
4-6	10 (22.2%)	0.201 ± 0.055	
7-10	32 (71.1%)	0.297 ± 0.014	
Uterine volumes, mm^3^			<0.001
50-100	4 (8.9%)	0.190 ± 0.013	
101-200	19 (42.2%)	0.258 ± 0.025	
>200	22 (48.9%)	0.312 ± 0.036	
Family history			0.912
Negative	21 (46.7%)	0.197 ± 0.011	
Positive	10 (22.2%)	0.203 ± 0.018	
Uncertain	14 (31.1%)	0.193 ± 0.037	
Undergo uterine surgery			0.588
Yes	34 (75.6%)	0.206 ± 0.024	
No	11 (24.4%)	0.197 ± 0.005	

Abbreviations: ADS: adenomyosis; PBAC: Pictorial Blood Loss Assessment Chart; VAS: visual analogue scale. ^∗^Data are presented as mean ± standard deviation.

## Data Availability

The data used to support the findings of this study are available from the corresponding author upon request.

## Supplementary Materials

Supplementary Materials Supplementary material including Figure S1, Figure S2, and Table S1 that are related to this article can be found in the online version. Figure S1: primary culture and identification of Eu_EEC and Eu_ESC. (S1A). Microscopic morphological features of primary Eu_EEC and Eu_ESC cultured for 72 hours. Scale bar: 200 *μ*m. (S1B). Anti-pan-cytokeratin and anti-vimentin antibodies were used to identify the specific marker of Eu_EEC and Eu_ESC, respectively. DAPI was used for nuclear counterstaining. The merged images exhibited the phenotypic characteristics of the corresponding cells. Scale bar: 100 *μ*m. Eu_EEC: adenomyotic eutopic endometrial epithelial cell; Eu_ESC: adenomyotic eutopic endometrial stromal cell. Figure S2: schematic of the regulation mechanism of circPVT1 in ADS. A schema chart was designed for summarizing and elucidating the proposed circPVT1/miR-145/Talin1 regulatory pathway in the etiopathogenesis of ADS.
