# Supplementary material for: Elevated Circular RNA PVT1 Promotes Eutopic Endometrial Cell Proliferation and Invasion of Adenomyosis via miR-145/Talin1 Axis
Source: Biomed Res Int. 2021 Feb 27;2021:8868700. doi: 10.1155/2021/8868700 (PMC7936912; doi:10.1155/2021/8868700)
Supplement: Supplementary Materials — Supplementary material including Figure S1, Figure S2, and Table S1 that are related to this article can be found in the online version. Figure S1: primary culture and identification of Eu_EEC and Eu_ESC. (S1A). Microscopic morphological features of primary Eu_EEC and Eu_ESC cultured for 72 hours. Scale bar: 200 μm. (S1B). Anti-pan-cytokeratin and anti-vimentin antibodies were used to identify the specific marker of Eu_EEC and Eu_ESC, respectively. DAPI was used for nuclear counterstaining. The merged images exhibited the phenotypic characteristics of the corresponding cells. Scale bar: 100 μm. Eu_EEC: adenomyotic eutopic endometrial epithelial cell; Eu_ESC: adenomyotic eutopic endometrial stromal cell. Figure S2: schematic of the regulation mechanism of circPVT1 in ADS. A schema chart was designed for summarizing and elucidating the proposed circPVT1/miR-145/Talin1 regulatory pathway in the etiopathogenesis of ADS. [file 8868700.f1.zip › TableS1.pdf]

Table S1. Information on the primers of specific genes in qRT-PCR analysis

Primer Name	Sequence(5′-3′)	Length(bps)
**circPVT1**		
Forward	5′CGACTCTTCCTGGTGAAGCATCTGAT3′	**26**
Reverse	5′TACTTGAACGAAGCTCCATGCAGC3′	**24**
**GAPDH**		
Forward	5′GGACCTGACCTGCCGTCTAG3′	**20**
Reverse	5′TAGCCCAGGATGCCCTTGAG3′	**20**
**Talin1**		
Forward	5′CTATATGCCACACCCGCCTC3′	**20**
Reverse	5′CCCAGGATTCCACGGGACTA3	**20**
**Actin**		
Forward	5′GCCGTGGTGGTGAAGCTGT3′	**19**
Reverse	5′ACCCACACTGTGCCCATCTA3′	**20**
**miR-145**		
Forward	5′GTCCAGTTTTCCCAGG3′	**16**
Reverse	5′GAGCAGGCTGGAGAA3′	**15**
**U6**		
Forward	5′CGCAAGGATGACACG3′	**15**
Reverse	5′GAGCAGGCTGGAGAA3	**15**
**miR-145 RT**	5′GTCGTATCCAGTGCAGGGTCCGAGG TA **TTCGCACTGGATACGACAGAACAGT3′**	**52**
