# Supplementary figures and images for: Elevated Circular RNA PVT1 Promotes Eutopic Endometrial Cell Proliferation and Invasion of Adenomyosis via miR-145/Talin1 Axis
Source: Biomed Res Int. 2021 Feb 27;2021:8868700. doi: 10.1155/2021/8868700 (PMC7936912; doi:10.1155/2021/8868700)

**A**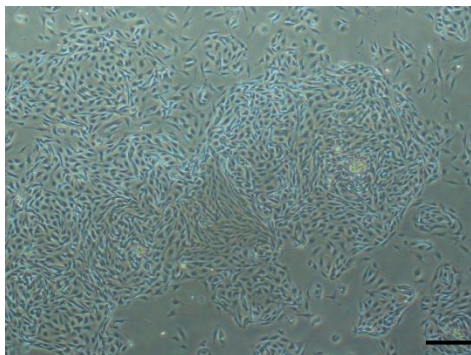

Eu\_EEC

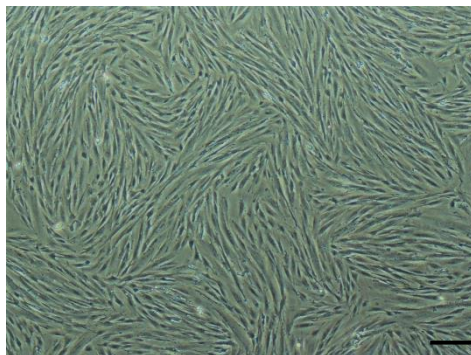

Eu\_ESC

**B**

Pan-cytokeratin

DAPI

Merge

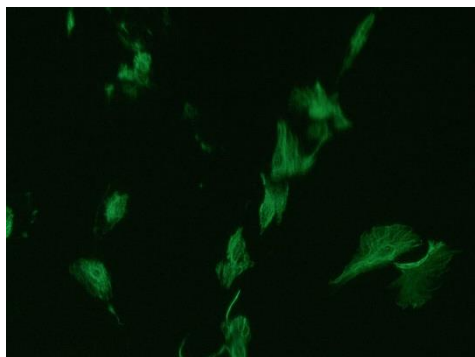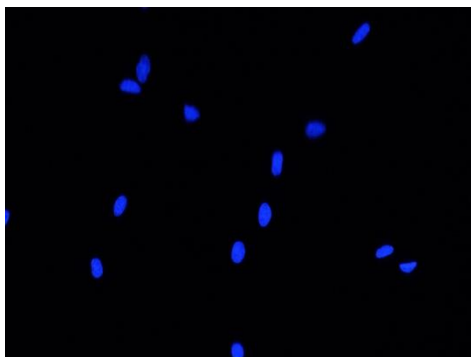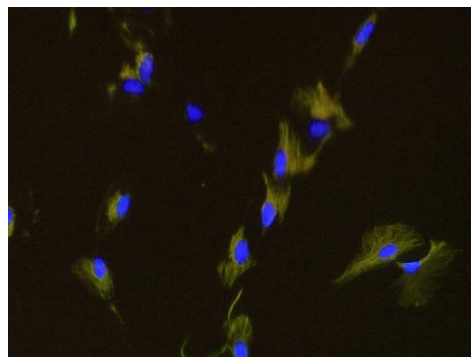

Eu\_EEC

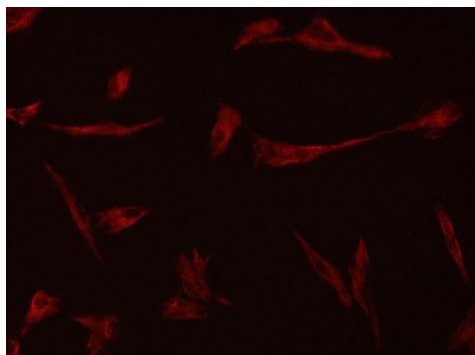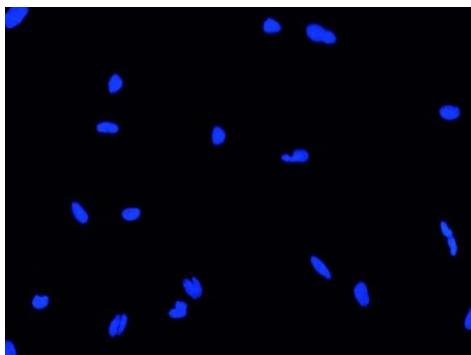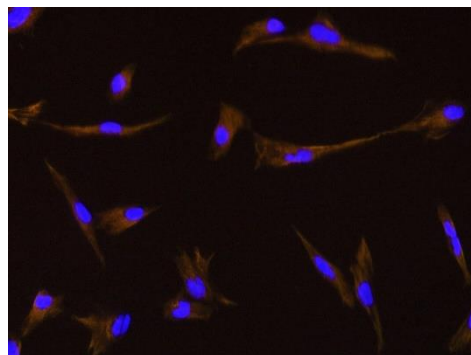

Eu\_ESC

Vimentin

DAPI

Merge

Supplement: Supplementary Materials — Supplementary material including Figure S1, Figure S2, and Table S1 that are related to this article can be found in the online version. Figure S1: primary culture and identification of Eu_EEC and Eu_ESC. (S1A). Microscopic morphological features of primary Eu_EEC and Eu_ESC cultured for 72 hours. Scale bar: 200 μm. (S1B). Anti-pan-cytokeratin and anti-vimentin antibodies were used to identify the specific marker of Eu_EEC and Eu_ESC, respectively. DAPI was used for nuclear counterstaining. The merged images exhibited the phenotypic characteristics of the corresponding cells. Scale bar: 100 μm. Eu_EEC: adenomyotic eutopic endometrial epithelial cell; Eu_ESC: adenomyotic eutopic endometrial stromal cell. Figure S2: schematic of the regulation mechanism of circPVT1 in ADS. A schema chart was designed for summarizing and elucidating the proposed circPVT1/miR-145/Talin1 regulatory pathway in the etiopathogenesis of ADS. [file 8868700.f1.zip › FiguresS1.pdf]

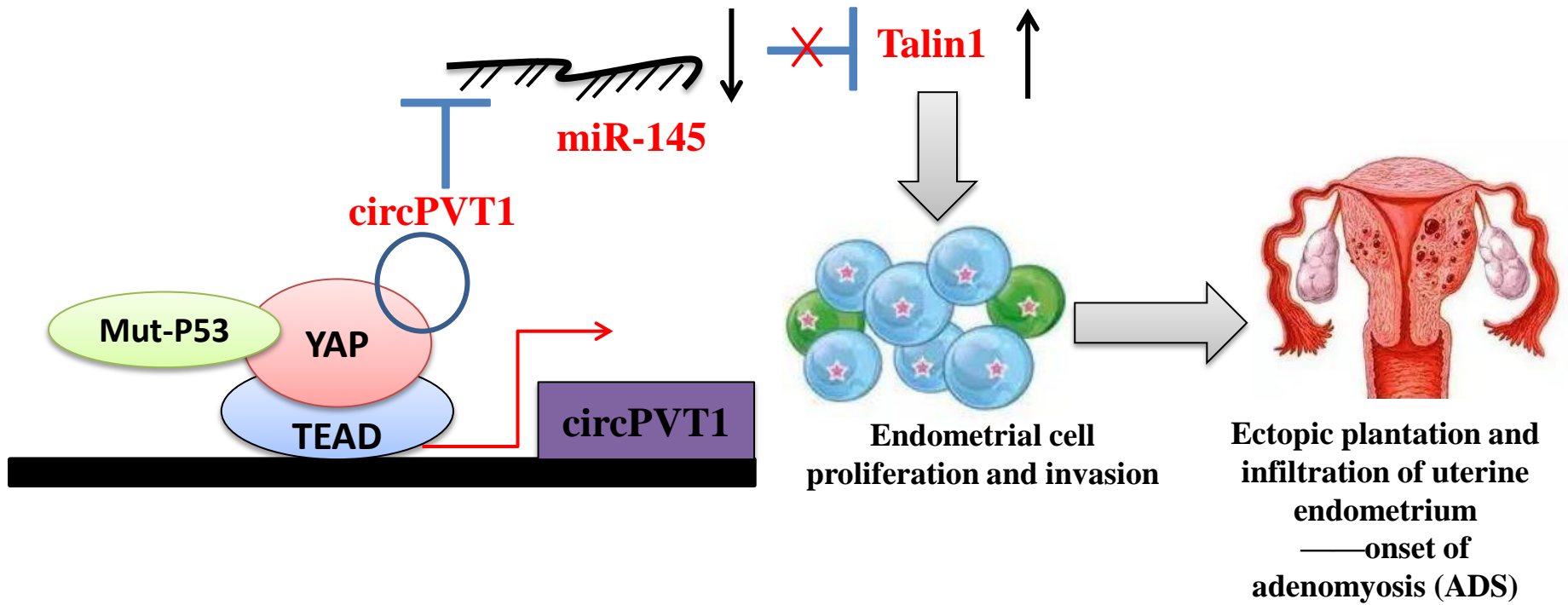

Supplement: Supplementary Materials — Supplementary material including Figure S1, Figure S2, and Table S1 that are related to this article can be found in the online version. Figure S1: primary culture and identification of Eu_EEC and Eu_ESC. (S1A). Microscopic morphological features of primary Eu_EEC and Eu_ESC cultured for 72 hours. Scale bar: 200 μm. (S1B). Anti-pan-cytokeratin and anti-vimentin antibodies were used to identify the specific marker of Eu_EEC and Eu_ESC, respectively. DAPI was used for nuclear counterstaining. The merged images exhibited the phenotypic characteristics of the corresponding cells. Scale bar: 100 μm. Eu_EEC: adenomyotic eutopic endometrial epithelial cell; Eu_ESC: adenomyotic eutopic endometrial stromal cell. Figure S2: schematic of the regulation mechanism of circPVT1 in ADS. A schema chart was designed for summarizing and elucidating the proposed circPVT1/miR-145/Talin1 regulatory pathway in the etiopathogenesis of ADS. [file 8868700.f1.zip › FiguresS2.pdf]
